# Topical Capsaicin in Poly(lactic-co-glycolic)acid (PLGA) Nanoparticles Decreases Acute Itch and Heat Pain

**DOI:** 10.3390/ijms23095275

**Published:** 2022-05-09

**Authors:** Nathalie M. Malewicz, Zahra Rattray, Sebastian Oeck, Sebastian Jung, Vicente Escamilla-Rivera, Zeming Chen, Xiangjun Tang, Jiangbing Zhou, Robert H. LaMotte

**Affiliations:** 1 Department of Anesthesiology, Yale University School of Medicine, 330 Cedar St, New Haven, CT 06510, USA; sebastian.jung@rub.de (S.J.); vicentee@arizona.edu (V.E.-R.); 2 Clinics for Anesthesiology, Intensive Care and Pain Medicine, Medical Faculty of Ruhr-University Bochum, BG University Hospital Bergmannsheil, 44789 Bochum, Germany; 3 Strathclyde Institute of Pharmacy and Biomedical Sciences, University of Strathclyde, Glasgow G4 0RE, UK; zahra.rattray@strath.ac.uk; 4 Department of Therapeutic Radiology, Yale University School of Medicine, New Haven, CT 06510, USA; sebastian.oeck@uk-essen.de; 5 Department of Medical Oncology, West German Cancer Center, University Hospital Essen, 45147 Essen, Germany; 6 ZEMOS Center for Solvation Science, Ruhr University Bochum, 44801 Bochum, Germany; 7 Department of Otolaryngology—Head and Neck Surgery, College of Medicine, The University of Arizona, Tucson, AZ 85724, USA; 8 Department of Neurosurgery, Yale University School of Medicine, New Haven, CT 06510, USA; czeming@hotmail.com (Z.C.); xiangjun.tang@yale.edu (X.T.); jiangbing.zhou@yale.edu (J.Z.)

**Keywords:** capsaicin, nanoparticles, human, pruritogens, desensitization, itch

## Abstract

Background: Capsaicin, the hot pepper agent, produces burning followed by desensitization. To treat localized itch or pain with minimal burning, low capsaicin concentrations can be repeatedly applied. We hypothesized that alternatively controlled release of capsaicin from poly(lactic-co-glycolic acid) (PLGA) nanoparticles desensitizes superficially terminating nociceptors, reducing burning. Methods: Capsaicin-loaded PLGA nanoparticles were prepared (single-emulsion solvent evaporation) and characterized (size, morphology, capsaicin loading, encapsulation efficiency, in vitro release profile). Capsaicin-PLGA nanoparticles were applied to murine skin and evaluated in healthy human participants (n = 21) for 4 days under blinded conditions, and itch and nociceptive sensations evoked by mechanical, heat stimuli and pruritogens cowhage, β-alanine, BAM8-22 and histamine were evaluated. Results: Nanoparticles (loading: 58 µg capsaicin/mg) released in vitro 23% capsaicin within the first hour and had complete release at 72 h. In mice, 24 h post-application Capsaicin-PLGA nanoparticles penetrated the dermis and led to decreased nociceptive behavioral responses to heat and mechanical stimulation (desensitization). Application in humans produced a weak to moderate burning, dissipating after 3 h. A loss of heat pain up to 2 weeks was observed. After capsaicin nanoparticles, itch and nociceptive sensations were reduced in response to pruritogens cowhage, β-alanine or BAM8-22, but were normal to histamine. Conclusions: Capsaicin nanoparticles could be useful in reducing pain and itch associated with pruritic diseases that are histamine-independent.

## 1. Introduction

A persistent itch that accompanies neurological, dermatological and systemic diseases can cause suffering and a loss in the quality of life [1]. While receptors for pruritogens are expressed in cutaneous nociceptors, there are few treatments that block the pathological transmission of pruritic information in these neurons in disorders causing acute or chronic itch. There are two types of pruriceptive nociceptors that transduce and transmit pruritic information from the skin [2,3,4]. One type responds to noxious mechanical and heat stimuli and to pruritogens that elicit a histamine independent itch [5]. These pruritogens include cysteine proteases, such as mucunain in the trichomes of cowhage, β-alanine, and bovine adrenal medulla 8-22 (BAM8-22) [5,6,7,8,9,10]. The other type of pruriceptive nociceptor is mechanically insensitive and responds to histamine [11,12,13]. In humans, monkeys and mice, pruriceptive nociceptors responsive to histamine or histamine-independent pruritogens typically express TRPV1, a non-selective cation channel responsive to capsaicin and noxious heat [14,15,16]. Thus, one treatment for itch that has shown some effectiveness is the topical application of capsaicin, which activates these nociceptors, leading to a longer lasting desensitization [17]. Commercially available topical preparations of capsaicin containing different concentrations have been used in the management of cutaneous neuropathic sensations including post-herpetic pain [18,19,20], brachioradial pruritus, and atopic dermatitis [21,22]. In an experimental study, daily topical application of capsaicin in humans elicited a persistent burning pain. After four days, there was a loss of transient heat pain and itch in response to cowhage spicules [23]. The itch to intradermal injection of histamine was unaffected by capsaicin. However, responses to β-alanine and BAM8-22 were not tested. In other studies, the transdermal delivery of higher concentrations of capsaicin, (e.g., 8% capsaicin in the Qutenza patch over 24 h) reduced or eliminated both histamine-dependent and histamine-independent itch and heat pain [24,25]. However, a major adverse event of capsaicin application experienced by patients is the painful burning sensation associated with the application of capsaicin. To treat localized itch or pain in humans through desensitization with low burning, very low concentrations of capsaicin are applied several times daily over a prolonged period of time. Strategies including the encapsulation of capsaicin in controlled release systems, such as poly(lactic-co-glycolic acid) nanoparticles (PLGA NPs) could reduce these adverse effects associated with capsaicin application. The slow-release effect of PLGA NPs as a carrier for bupivacaine or opioids to reduce nociceptive behavior in mice has been studied previously [26,27,28].

In the present study, we test the hypothesis that at single application of controlled release capsaicin-loaded PLGA NPs, over a period of hours to days, can achieve desensitization to histamine-independent pruritogens and to transient noxious heat, while eliciting a minimal ongoing burning pain sensation.

## 2. Results

### 2.1. Physicochemical Attributes of NPs

**Figure 1:**
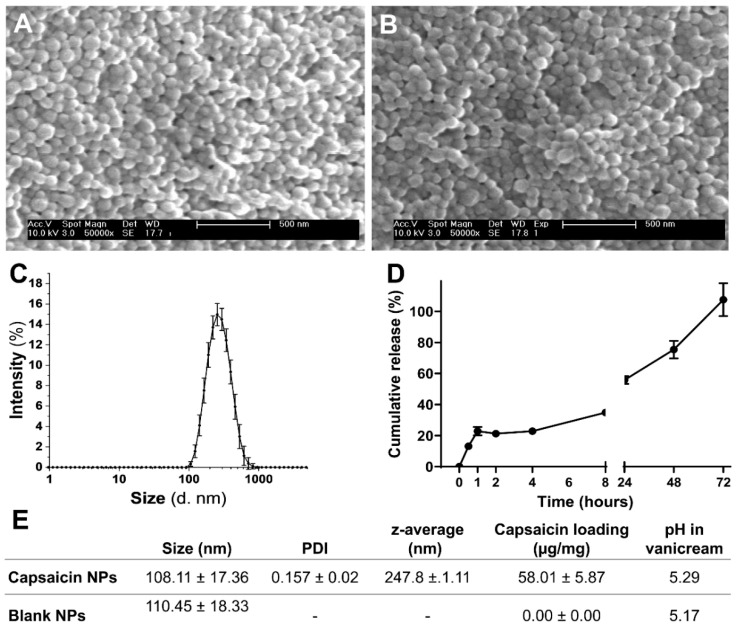
Characterization of the physicochemical attributes of NPs. (**A**) SEM of capsaicin NPs. (**B**) SEM of blank NPs. Scale bar is 500 nm. (**C**) Intensity-based particle size distribution as measured by dynamic light scattering. (**D**) Cumulative in vitro release of capsaicin from NPs over time under agitation. (**E**) Measurements of size, capsaicin loading, encapsulation efficiency and pH (in Vanicream) for capsaicin-loaded and blank NPs. Mean ± SEM.

SEM analysis showed that both blank and capsaicin PLGA NPs are spherical in morphology and uniform in size (Figure 1A,B). All NPs were spherical in morphology, with a corresponding diameter of 108.11 ± 17.36 and 110.45 ± 18.33 nm as determined by analysis of SEM micrographs. No statistically significant difference in size was noted between blank and capsaicin NPs (Figure 1E). Data obtained from the dynamic light scattering analysis of capsaicin NP intensity-based particle size distribution showed a unimodal size distribution (Figure 1C), with a corresponding peak occurring at approximately 250 nm and a polydispersity index of 0.157. The zeta potential for capsaicin PLGA NPs was determined as −17.53 ± 0.13 mV.

Capsaicin drug loading and encapsulation efficiencies were determined using a capsaicin ELISA kit. Capsaicin loading was determined to be 58.01 ± 5.87 µg per 1 mg for capsaicin-loaded PLGA NPs, and 0 µg per 1 mg of blank PLGA NPs. Therefore, capsaicin loading was measured as 5.8% by weight, and the corresponding encapsulation efficiency was determined to be 16.57%.

In vitro capsaicin release from PLGA NPs under agitation showed an initial capsaicin release of approximately 22.9% from the NPs within the first hour (Figure 1B). This initial burst, leading to a release of 34% of the payload at 8 h, of capsaicin release from PLGA NPs continually increased until reaching a peak at about 72 h (Figure 1D).

The pH of Vanicream (5.28) was not changed by the addition of capsaicin-loaded (5.29) and blank PLGA NPs (5.17) (Figure 1E). The resultant pH was appropriate as to minimize pH-related irritation following topical application.

### 2.2. In Vivo Quantification of Capsaicin

The amount of capsaicin in murine cheek skin after 24 h of treatment with capsaicin NPs was measured as 0.16 µg/mL in 6 µg/µL total protein. In comparison, skin in which 30 µg of capsaicin was intradermally injected before harvesting exhibited a slightly higher amount of 0.25 µg/mL. In contrast, the amount of capsaicin in the skin treated with blank NPs was 0 µg/mL.

**Figure 2:**
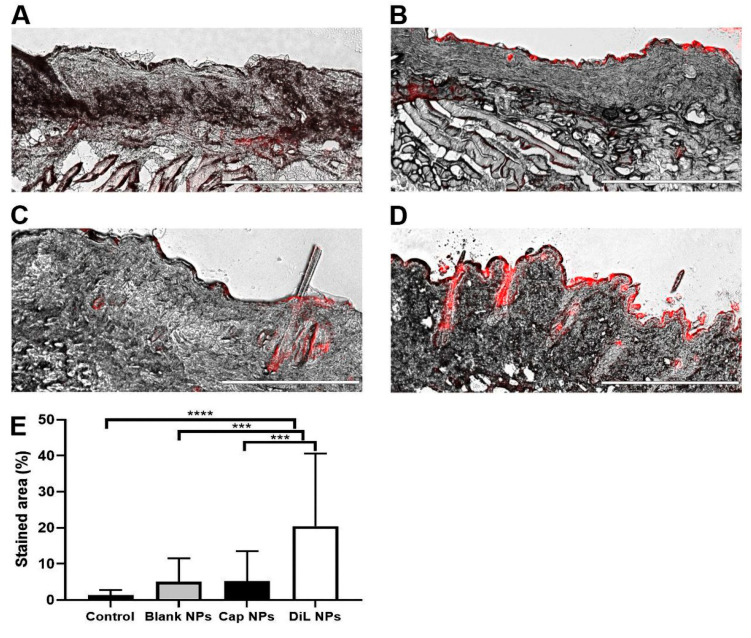
Intracutaneous penetration of NPs topically applied to the skin in the mouse. Exemplary microscope images (Scale bar is 400 µm) of (**A**) a potential autofluorescence (red area) of untreated skin from mouse cheek, (**B**) after treatment with blank NPs, (**C**) after treatment with capsaicin NPs, (**D**) treated with red dye (red area) containing DiL NPs. (**E**) Mean values of red color measured in cheek tissue from 18 samples per group. *** *p* < 0.001, **** *p* < 0.0001, mean ± SD, one-way ANOVA with Bonferroni correction.

The in vivo skin penetration ability of capsaicin NPs containing red dye into the skin of the cheek was also assessed. The red colored areas were measured relative to the unstained skin. Untreated control tissue had only small amounts of red stain detected in 1 of 18 samples in the dermis and 3 of 18 in the epidermis, most likely due to autofluorescence of high-density areas of the tissue (Figure 2A). Blank NPs and capsaicin NPS (Figure 2B,C) showed insignificantly higher amounts of red stain in the dermis and epidermis possibly due to agglomerated NPs. The amount of red stain after DiL NPs was detectable in all samples in the epidermis and in 17 of 18 samples in the dermis, mainly located in more superficial regions (Figure 2D). The amount of staining for the DiL NPs was significantly higher than in control tissue or tissue exposed to blank or capsaicin NPs (Figure 2E). This finding is consistent with a good penetration with NPs.

### 2.3. Behavioral Responses of Mice to Mechanical and Heat Stimuli

**Figure 3:**
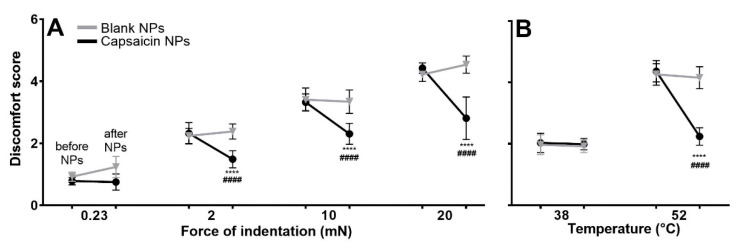
Discomfort scores to mechanical and heat stimuli applied to the cheek. The mechanical stimuli were produced by application of von-Frey type filaments with indentation forces (and tip diameters) of 0.23 mN (67 µm) and 2, 10 and 20 mN (100µm). Heat stimuli were applied by a thermode preheated to the indicated temperature. Each stimulus was applied prior to and 24 h following removal of NPs. (**** *p* < 0.0001) for significance before and after treatment, #### indicating significant differences between the blank vs. capsaicin NPs groups *p* < 0.0001, error bars: SD n = 10 male mice per group. RMANOVA—2 within timepoints (before, after treatment) × treatments (capsaicin vs. blank NPs) × forces (0.23, 2, 10, 20 mN) for (**A**) and temperatures for (**B**) (38, 52 °C) with Bonferroni correction.

Mice were scored for aversive behavioral responses to punctate mechanical indentation and heat stimulation of the cheek prior to and 24 h after application of a Finn chamber containing either blank NPs or capsaicin-loaded NPs. In comparison with scores obtained before NP application, mice receiving capsaicin NPs exhibited significantly lower discomfort scores to filament indentations of 2, 20 and 20 mN and to the heat stimulus of 52 °C Figure 3A,B). Responses to the lowest filament force of 0.23 mN and to the heat stimulus of 38 °C remained unchanged. In contrast, discomfort scores remained the same in response to each force of indentation and stimulus temperature before vs. after the application of blank NPs.

For human subjects, the highest indentation force and the heat stimulus of 50 °C, but not the two lowest forces or 39 °C stimulus, readily elicited pain when applied to the volar forearm. Thus, capsaicin NPs decreased the aversive behavior of mice to stimuli that are nociceptive for humans (Figure 3B).

### 2.4. Evaluation of the Sensations Evoked by Treatment with Capsaicin PLGA NPs in Humans

**Figure 4:**
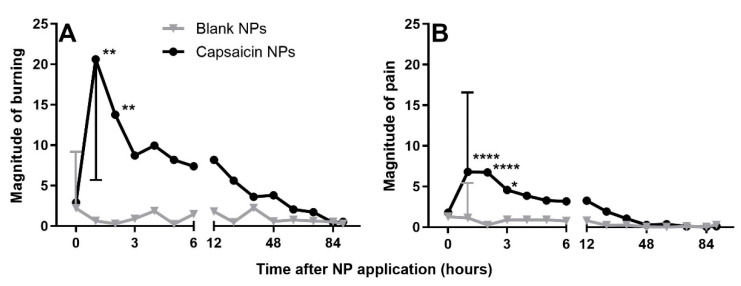
Magnitude ratings of spontaneous (background) sensations of burning and pain referred to the sites of application of blank and capsaicin filled NPs. (**A**) Magnitude of burning, (**B**) magnitude of pain. Mean ± SD, n = 21, mixed effect model 2 within subject treatments (capsaicin vs. placebo treatment) × 14 repeated timepoints (0–90 h) with Bonferroni correction. * *p* < 0.05, ** *p* < 0.01, **** *p* < 0.0001 for blank vs. NP comparison.

Subjects used the generalized labeled magnitude scale [10,29,30] to rate the average perceived intensity of any burning, pricking/stinging and pain sensation originating from beneath each Finn chamber following application of NPs. Subjects made ratings every hour for 6 h followed by every 12 h from 12 to 84 and a final rating at 90 h. Between 1 and 3 h after application of the chambers, subjects reported significantly higher rating scores from capsaicin NPs, in comparison to placebo (blank) NPs for burning, described as moderate, and pain rated as barely detectable to weak on the scale (Figure 4). These sensations subsided after 3 h and mirrored the in vitro measurements reflecting the initial burst of capsaicin release from NPs. After 3 h there were no significant differences in ratings of any sensory quality or in pain for the two sites of NP application. Although there were more frequent reports of pricking/stinging sensation but not itch in response to capsaicin during the first 12 h (Fishers Exact Test, *p* = 0.01), these sensory qualities did not yield significant differences in magnitude for the two treatments.

### 2.5. Effects of Capsaicin NPs on Thresholds and Magnitude Estimates of Mechanical and Heat Stimuli

**Figure 5:**
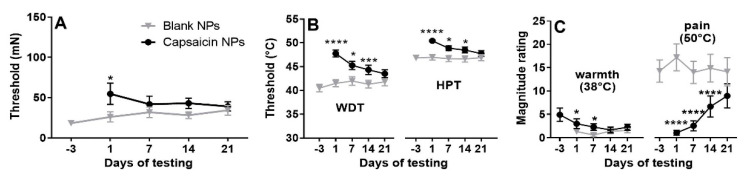
Mechanical pain thresholds, and thresholds and magnitude ratings of warmth and heat pain. (**A**) Thresholds for mechanically evoked pain (MPT) obtained at sites of blank and capsaicin NPs obtained 4 days before and on each designated day after removal of the NPs. (**B**) Detection thresholds for warmth (WDT) and heat pain (HPT, same format as in A). (**C**) Magnitude ratings of warmth and heat pain produced by 38 and 50 °C, respectively. n = 21, mixed effect model 2 within subject treatments (capsaicin vs. placebo treatment) × 4 repeated timepoints (0, 7, 14, 21 days) with Bonferroni correction. Means with SEMs for capsaicin and blank NPs are black and gray, respectively. * *p* < 0.05, *** *p* < 0.001 and **** *p* < 0.0001 for blank vs. NP comparison.

After removal of NP Finn chambers, mechanical pain thresholds for nociceptive sensations evoked by von Frey filaments were significantly higher for capsaicin in comparison to blank NP sites, but only on day 1 (Figure 5A).

The dominant sensory quality evoked by 50 °C was burning. The thresholds for detecting warmth (WDT) and painful heat (HPT) at the site of blank NPs administration were stable over the duration of the experiment. WDT and HPT increased on day 1 (after removal of Finn chambers with NP) in relation to prior tests after application of capsaicin NPs (Figure 5B). At this time, all subjects had a higher WDT and HPT on day 1 after capsaicin NP application in comparison with pre-treatment values at that site. A 2 treatment × 4 days mixed effect model followed by Bonferroni correction revealed a significant interaction between treatment and days (WDT: F(2.423, 48.46) = 6.976, *p* = 0.0012; HPT: F(2.651, 53.02) = 5.932, *p* = 0.0022). Both WDTs and HPTs were significantly higher at the capsaicin than blank NP site on days 1, 7 and 14. Recovery of WDT and HPT was observed on day 21.

There were also decreased magnitude estimates of the intensity of suprathreshold warming and noxious heat. Magnitude ratings of the warmth evoked by sustained warming at 38 °C on the capsaicin vs. blank sites occurred on days 1 through 14 (F = 9.657, *p* = 0.0055). Pain in response to 50 °C heat for 7 s as suprathreshold stimulation was reduced in all subjects and abolished in 16 of 21. The mean magnitude estimate of pain elicited by application of 50 °C heat was significantly lower on the capsaicin-treated side on days 1 through 14 (all *p* < 0.0001) but partially recovered on day 21 (*p* = 0.087); partial recovery was obtained (F = 23.70, *p* < 0.0001).

Noxious heat stimulation resulted in a local redness of the skin in 12 of 21 subjects before treatment and on the control site but in only one subject on the capsaicin-treated area. Another one of the capsaicin-treated subjects exhibited a white or vasoconstricted reaction. These findings suggest that capsaicin decreased heat-induced vascular dilation.

In contrast to the effects of capsaicin in decreasing the perceived intensity of warmth and heat pain, a RMANOVA revealed no significant differences between NPs and blank NPs sites in subjective magnitude estimates of mechanically evoked pain for any force on any day of testing (Supplementary Figure S1).

### 2.6. Effects of Capsaicin NPs on Itch, Nociceptive Sensations and Dysesthesias Evoked by Pruritogens

**Figure 6:**
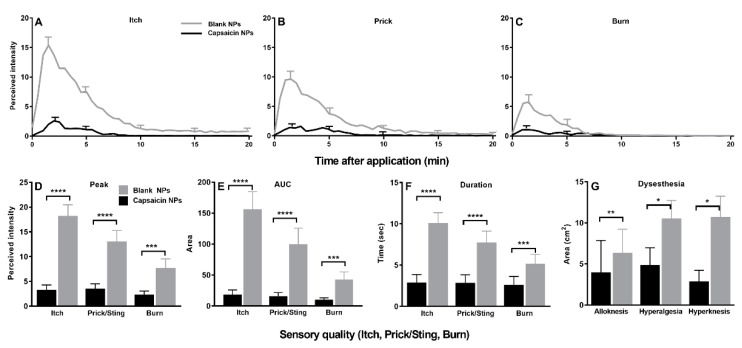
Effects of capsaicin NPs pretreatment on cowhage evoked sensations and dysesthesias applied by spicules. (**A**–**C**) Time course of perceived intensities of itch, pricking/stinging, and burning. Data are means with SEMs. For clarity, the SEMs are presented at the peak and every 5 min thereafter. (**D**) Peak magnitude (Peak) of each sensory quality. (**E**) Area under the rating curve (AUC). (**F**) Duration of response. (**G**) Area of each type of dysesthesia: alloknesis, hyperalgesia and hyperknesis. n = 21, (**A**–**C**) repeated measures two-way ANOVA; two repeated treatment groups (capsaicin vs. placebo treatment) × 40 repeated timepoints (0–20 min) with Bonferroni correction, (**D**–**G**) Wilcoxon test. * *p* < 0.05, ** *p* < 0.01, *** *p* < 0.001 and **** *p* < 0.0001.

In response to cowhage application on the day of NP removal, there was a significant decrease in the peak magnitude, AUC, and duration of itch and each type of nociceptive sensation on the capsaicin vs. the blank NP site (Figure 6A–F). Eight subjects reported a complete abolition of itch as well as heat pain. In accordance with a decrease in sensations, capsaicin NPs also significantly decreased the areas of alloknesis, hyperalgesia and hyperknesis induced by cowhage (Figure 6G).

**Figure 7:**
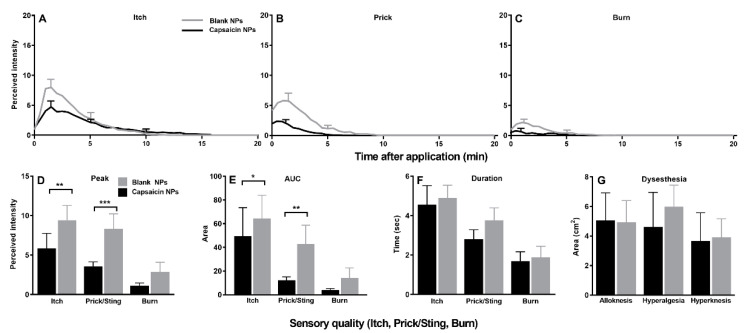
Effects of capsaicin NP pretreatment on sensations and dysesthesias evoked by injection of beta alanine. (**A**–**C**) Time course of perceived intensities of itch, pricking/stinging and burning. Same format as Figure 7. (**D**) Peak magnitude (Peak) of each sensory quality. (**E**) Area under the rating curve (AUC). (**F**) Duration of response. (**G**) Area of each type of dysesthesia: alloknesis, hyperalgesia and hyperknesis. n = 21, (**A**–**C**) repeated measures two-way ANOVA; two repeated treatment groups (capsaicin vs. placebo treatment) × 40 repeated timepoints (0–20 min) with Bonferroni correction, (**D**–**G**) Wilcoxon test. Data are means with SEMs. * *p* < 0.05 and ** *p* < 0.01and *** *p* < 0.001.

In response to injections of β-alanine, the perceived intensities of itch and each nociceptive sensation were reduced early after injection, starting at 1 min after injection and lasting 2.5 min for itch and 3.5 min for the nociceptive sensations (Figure 7A–C). In relation to placebo (blank) NPs, capsaicin NPs resulted in significantly lower peak magnitude ratings and AUCs for both itch and pricking/stinging sensations but not for burning (Figure 6D,E). In contrast, there were no significant differences in the duration of each quality of sensation (Figure 7F). There was a significant decrease in the area of hyperalgesia (Figure 7G), and no significant differences in areas of alloknesis or hyperknesis between the capsaicin and blank NP sites were reported.

**Figure 8:**
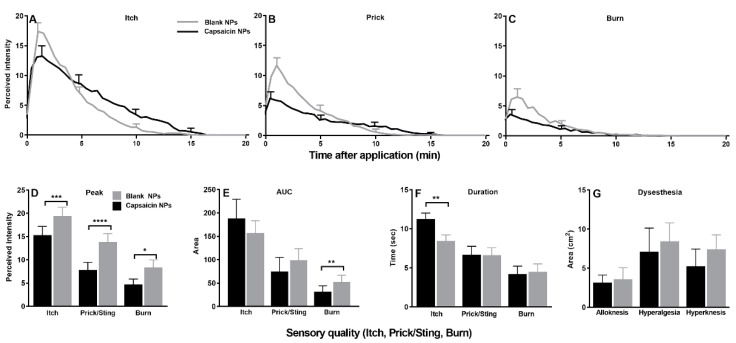
Effects of capsaicin NP pretreatment on sensations and dysesthesias evoked by injection of BAM8-22. (**A**–**C**) Time course of perceived intensities of itch, pricking/stinging, and burning. Same format as Figure 7. (**D**) Peak magnitude (Peak) of each sensory quality. (**E**) Area under the rating curve (AUC). (**F**) Duration of response. (**G**) Area of each type of dysesthesia: alloknesis, hyperalgesia and hyperknesis. n = 21, (**A**–**C**) repeated measures two-way ANOVA; two repeated treatment groups (capsaicin vs. placebo treatment) × 40 repeated timepoints (0–20 min) with Bonferroni correction, (**D**–**G**) Wilcoxon test. Data are means with SEMs. * *p* < 0.05, ** *p* < 0.01, *** *p* < 0.001 and **** *p* < 0.0001.

In response to injections of BAM8-22, the perceived intensities of itch and each nociceptive sensation were significantly lower for capsaicin in comparison with blank NP application (Figure 8A–C). The reduced ratings at the capsaicin application site were apparent within the first minutes after injection. Sensation at the capsaicin application site was reduced in comparison to blank for up to 3 min after injection for itch, up to 7 min for pricking/stinging and 5 min for burning. Similar to the effects of β-alanine, the peak ratings were significantly lower at the capsaicin site for each sensory quality (Figure 8D).

In contrast to β-alanine, for injection of BAM8-22 the AUC was lower only for pricking/stinging and the duration of itch was significantly longer for the capsaicin site (Figure 8E,F). Differences in the areas of dysesthesia did not reach significance (Figure 8G). The only skin reaction to be affected was the area of local redness which was smaller on the capsaicin than the blank NP site (1.2 ± 2.2 vs. 5.4 ± 7.5 cm^2^, *p* = 0.0005).

**Figure 9:**
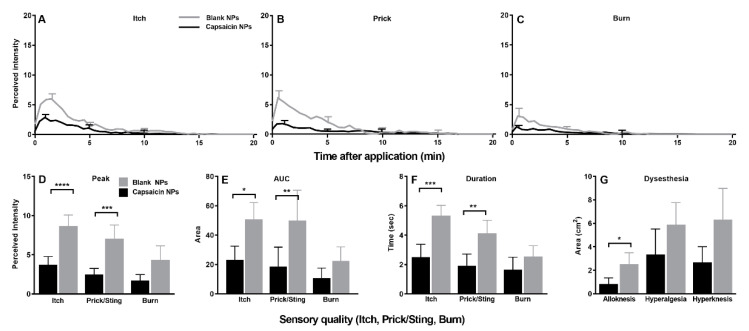
Effects of capsaicin NP pretreatment on sensations and dysesthesias evoked by BAM8-22 spicules. (**A**–**C**) Time course of perceived intensities of itch, pricking/stinging, and burning. Same format as Figure 7. (**D**) Peak magnitude (Peak) of each sensory quality. (**E**) Area under the rating curve (AUC). (**F**) Duration of response. (**G**) Area of each type of dysesthesia: alloknesis, hyperalgesia and hyperknesis. n = 21, (**A**–**C**) repeated measures two-way ANOVA; two repeated treatment groups (capsaicin vs. placebo treatment) × 40 repeated timepoints (0–20 min) with Bonferroni correction, (**D**–**G**) Wilcoxon test. Data are means with SEMs. * *p* < 0.05, ** *p* < 0.01, *** *p* < 0.001 and **** *p* < 0.0001.

The effects of capsaicin on responses to spicules containing BAM8-22 were similar to those of cowhage, resulting in significantly lower peak ratings, AUCs and shorter durations of itch and pricking/stinging in comparison with the effects of blank NPs (Figure 9A–F). However, unlike cowhage there were no differences in the peak magnitude, rating, or duration for burning. Treatment with capsaicin NPs provoked a reduction in alloknesis induced by BAM8-22 spicules (Figure 9G). There was also a decrease in the area of local redness at the application site within the capsaicin-treated area (0.8 ± 2.9 vs. 0.1 ± 0.1 cm^2^, *p* = 0.044).

Capsaicin NPs did not affect the maximum perceived intensity or duration of the histamine injection-evoked itch or pricking stinging compared to the blank NPs (Supplementary Figure S2). Interestingly, 17 of 21 subjects exhibited a blanching of the skin, resembling a vasoconstriction around the histamine injection site. This effect was only observed on capsaicin-treated sites. In addition, treatment of capsaicin NP reduced the area of the local redness (27.2 ± 15.7 vs. 20.7 ± 19.0 cm^2^, *p* = 0.0239). No significant differences were observed in areas of histamine-induced alloknesis, hyperalgesia and hyperknesis.

Following the application of histamine spicules, the duration of each sensory quality was slightly but significantly decreased at the capsaicin vs. blank site without any change in peak magnitude (Supplementary Figure S3). The areas of hyperalgesia and hyperknesis were significantly smaller at the capsaicin vs. blank site (7.657 ± 13.09 vs. 4.769 ± 9.13 cm^2^, *p* = 0.0408) and (4.982 ± 7.367 vs. 1.435 ± 2.792 cm^2^, *p* = 0.0136), respectively. The area of local redness was also significantly decreased on the capsaicin site (7.0 ± 5.6 vs. 1.5 ± 2.9 cm^2^, *p* = 0.0003) and the appearance of white areas around the capsaicin application area was observed in 10 out of 21 subjects.

Capsaicin had the major effect of reducing and in some instances eliminating the itch and nociceptive sensations evoked by the histamine-independent pruritogens cowhage, β-alanine and BAM8-22 but had little or no significant effect on the sensations evoked by histamine.

## 3. Discussion

In this study, capsaicin-loaded PLGA NPs were developed for topical application in humans and their in vitro physicochemical attributes were characterized. An initial in vitro release of 22.9% capsaicin within the first hour was observed (burst), followed by a lower continuous release until 72 h, where complete capsaicin release was observed. In a murine model, capsaicin NPs applied to the skin for 24 h penetrated the dermis and led to a behavioral desensitization to heat and mechanical stimuli. Application in humans caused weak to moderate burning sensation lasting approximately 2 h followed by a short-lasting elevation of mechanical pain threshold and a lasting desensitization to warm and noxious heat. During this period of heat desensitization there was also a significant decrease in the magnitude ratings of itch and nociceptive sensations evoked by cowhage, β-alanine, and BAM8-22 but little or no significant effect on sensations elicited by histamine.

PLGA NPs to alter pain or itch in humans have not yet been applied in practice [28]. Capsaicin-loaded PLGA NPs have been investigated for the treatment of chronic pain in animal models, due to their controllable prolonged release and biocompatibility and acceptable safety [26,31,32]. Our prototype PLGA NPs showed an initial low burst effect and a continuous release profile designed to desensitize while diminishing an unpleasant burning sensation. Using other PLGA compositions or biocompatible polymers to encapsulate capsaicin could alter the release profile [33]. 

Capsaicin has been applied in previous studies to desensitize the skin to noxious and pruritic stimuli experimentally applied to the skin in human volunteers. For example, three days of repeated topical applications of 0.1% capsaicin abolished transient heat pain and itch to cowhage but not itch to histamine [23]. A higher dose of 8% topically applied capsaicin was subsequently found to reduce both histaminergic and non-histaminergic cowhage itch [25].

A limitation of other topical applications of capsaicin is the associated prolonged burning pain sensation. Conversely, the slow release of the capsaicin from NPs in the present study leads to low pain and burning sensation. A positive control with active capsaicin was not applied in this study. However, lower burning pain was observed in comparison, for example to previously described for a 1 or 24 h application of 8% capsaicin in other studies [24,25] or capsaicin injection [34]. Therefore, capsaicin NP formulations could be a valuable treatment application for pain- and itch-associated diseases. The missing desensitization to histamine in our study could be due to the late testing after desensitization, when some functions had almost recovered. Other reasons could be the small area of desensitization which, after injection, was smaller than the wheal, indicating that the histamine diffused beyond the capsaicin-treated area into surrounding untreated skin. This was less likely to happen with the histamine spicules, whose effects were confined within the treated area. An effect of capsaicin after histamine injection was especially seen in a localized vasoconstriction or white area in the capsaicin application area surrounded by a local redness [24,35,36]. Other possibilities would be the amount of capsaicin being too low or the penetration depth not deep enough to reach the terminal endings of histamine-responsive mechanically insensitive nociceptors.

Capsaicin application decreased sensory responses to β-alanine and BAM8-22 spicules thought to activate mechanosensitive polymodal nociceptors mediating histamine-independent reactions [7,8,14,37,38]. Consequently, capsaicin could be effective in treating chronic pruritic disorders that elicit histamine-independent itch. Additionally, the capsaicin NP formulation is a feasible approach which should be investigated in further studies for its efficacy and safety to treat chronic pain and itch.

Experimental outcomes from the present work are clinically relevant and demonstrate the novel concept of controlled NP delivery of capsaicin for the future treatment of chronic, localized itch or pain that typically accompanies eczema or post-herpetic neuralgia. PLGA is readily hydrolyzed to its monomer components (lactic acid and glycolic acid), which are metabolized to non-toxic byproducts, with a demonstrated safety profile. Numerous FDA-approved PLGA formulations are routinely used in the clinical setting [39]. We anticipate that a single topical application of capsaicin-loaded NPs would minimize sensory discomfort and the need for multiple clinic visits for re-application. The minimization of discomfort would increase compliance of patients using the preparation. Thus, the present findings could motivate the use of capsaicin NPs for the clinical treatment of certain types of chronic inflammatory or neuropathic itch.

## 4. Materials and Methods

### 4.1. Nanoparticle Preparation

Capsaicin-loaded PLGA nanoparticles (NPs) were prepared under clean conditions using a single-emulsion, solvent evaporation approach previously described by Zhou et al. [26,32]. The PLGA (50:50 Poly(DL-lactide-co-glycolide)) and capsaicin (Sigma Aldrich, St. Louis, MO, USA) were mixed and dissolved in ethyl acetate (60 mg of capsaicin per 100 mg PLGA). The resultant solution was added dropwise to 5% polyvinyl alcohol (PVA). Following sonication, the resultant mixture was slowly added to a 0.3% *v*/*v* PVA solution while stirring. Over 5 to 6 h, the ethyl acetate was allowed to evaporate under a fume hood. Capsaicin-loaded PLGA NPs were collected through centrifugation (18,000× *g* for 20 min) and washed twice in sterile de-ionized water to remove unencapsulated capsaicin. Following the final wash step, the NPs were collected and resuspended in 5 mL sterile de-ionized water. Trehalose was added as a cryoprotectant to the NP suspension at a mass ratio of 0.1:1 (trehalose:NP). After flash freezing with liquid nitrogen, the NPs were lyophilized for 2 days using a freeze dryer (Labconco, Kansas City, MO, USA). The dry NP powder was stored at −20 °C until further use. Blank NPs (placebo control) were prepared under identical conditions in the absence of capsaicin.

### 4.2. Analysis of NP Physicochemical Attributes

Scanning Electron Microscopy (SEM): The morphology and size of NPs were characterized by SEM. NPs were mounted on a carbon tape and sputter-coated with gold in an argon atmosphere using a sputter current of 40 mA (Dynavac Mini Coater; Dynavac, Hingham, MA, USA). SEM analysis was carried out with a Philips XL30 SEM using a LaB electron gun with an accelerating voltage of 3 kV. NP size was determined from SEM micrographs using ImageJ software (version 1.5, National Institutes of Health (NIH), Bethesda, MD, USA).

Dynamic Light Scattering: Corresponding particle size distribution (hydrodynamic diameter) and polydispersity index were measured using a Zetasizer nano (Malvern, UK). The zeta potential of manufactured capsaicin NPs was measured using the electrophoretic light scattering function of the Zetasizer. All measurements were performed in triplicate.

### 4.3. Quantification of Drug Loading and Encapsulation Efficiency

A total of 1 mg of PLGA NPs was dissolved in 100 µL DMSO and made to a volume of 1 mL by adding PBS. An Enzyme-linked Immunosorbent Assay (ELISA) Capsaicin High Sensitivity Plate Kit (Beacon Analytical Cat.# 20-0027, Saco, ME, USA) was used for the quantification of capsaicin content as per manufacturer instructions. In brief, a serial dilution of PLGA NPs and a capsaicin standard for a calibration curve was made. Aliquots of 100 µL volume were pipetted in triplicates into the wells of antibody-coated stripes, mixed with 100 µL of enzyme conjugate and incubated for 30 min under agitation. Subsequently, the wells were washed five times with water, incubated with 100 µL reaction substrate for 10 min, and the reaction was stopped by adding 100 µL stop solution to each well. The resultant absorbance (450 nm–650 nm) was measured by a plate reader. The amount of capsaicin in the resultant solution was determined from the calibration curve, and the corresponding NPs loading (Equation (1)) and encapsulation efficiency (Equation (2)) were determined from the following equations.

(1) $$\;\mathrm{NP}\;\mathrm{capsaicin}\;\mathrm{loading}=\frac{\mathrm{Released}\;\mathrm{Capsaicin}\;\left(\mathrm{mg}\right)}{\mathrm{NP}\;\mathrm{weight}\;\left(\mathrm{mg}\right)}\times 100$$

(2) $$\mathrm{Encapsulation}\;\mathrm{Efficiency}=\frac{\mathrm{Measured}\;\mathrm{capsaicin}\;\mathrm{content}\;\left(\mathrm{mg}\right)}{\mathrm{Added}\;\mathrm{capsaicin}\;\mathrm{weight}\;\left(\mathrm{mg}\right)}\times 100$$

### 4.4. In Vitro Release of Capsaicin from PLGA Nanoparticles

The vitro release of capsaicin from NPs was evaluated at various time points in phosphate buffer (pH 6.5) at 35 °C in an agitated system (150 rpm). Aliquots were removed at pre-determined time points and replaced with the same volume of fresh pre-warmed phosphate buffer. Sample aliquots removed were centrifuged at 18.000 rpm for 10 min and the resultant supernatant was removed for the quantification of released capsaicin using the aforementioned ELISA capsaicin quantification kit. Cumulative in vitro release of capsaicin from PLGA NPs was determined for each timepoint.

### 4.5. Quantification of pH

The pH of the combination of cream and NPs for topical application was determined in triplicate, on the same formulation composition used for the topical application. Briefly, a 1 mg aliquot of capsaicin-loaded PLGA NPs was dissolved in 20 µL of sterile water, vortexed for 1 min and sonicated for 5 min. The resultant NP suspension and pure sterile water were added to 80 mg of Vanicream dissolved in 50 µL DMSO and mixed in 15 mL of water. The resultant pH was quantified after 1 hr using a pH meter. The same procedure was carried out for the placebo (blank) NPs.

### 4.6. Preparation of the NP Cream for Topical Application

A 1 mg aliquot of NPs was suspended in 20 μL of sterile water by vortexing for 1 min, and then placed in an ultrasonic bath for 5 min. The resultant NP suspension was stirred into 80 mg of Vanicream. Then resultant preparation was filled into a 12 mm Finn chamber for human subjects. An equivalent dose corresponding to 1/8 of the human total dose was filled into an 8-mm Finn-type chamber for mice. Placebo controls included Vanicream and blank PLGA NP mixtures.

### 4.7. In Vivo Application of Capsaicin NPs in a Murine Model

#### 4.7.1. Animals

C57BL/6 male mice (Charles River Laboratories), each weighing 20–25 g and aged 6–8 weeks old, were used for behavioral experiments, ultrasound, skin thickness measurements and histological evaluation. All experimental procedures were approved by the Institutional Animal Care and Use Committee of Yale University School of Medicine and were in accordance with guidelines provided by the National Institute of Health and the International Association for the Study of Pain. Mice were housed under a 12 h light/dark cycle with free access to standard laboratory food and water.

#### 4.7.2. In Vivo Application of Capsaicin NPs

The Finn-like chambers for mice were constructed in the Yale Medical School Machine Shop from polyether ether ketone (PEEK) (Trident Plastics, Ivyland, PA, USA). The chamber had an outside diameter of 8 mm and a well that was 5 mm wide and 0.2–0.3 mm deep. PEEK is a medically compatible plastic and used for orthopedic surgery, bone replacement, dental implants, and other biomedical applications [40]. The chamber accommodated up to 10 mg of Vanicream filled with NPs in the same concentration as used for humans. This amount contained a maximal amount of 44 µg of capsaicin. The dermal LD50 for capsaicin was determined to be > 512 mg/kg in mice [41].

Under brief anesthesia (2% isoflurane in 300 mL/min oxygen), the PEEK chamber was applied on the shaved cheek of the mouse with a thin coat of cyanoacrylate glue on the rim. An Elizabethan collar prevented the animal from removing the cup for 24 h. After this time, the chamber was removed with a small amount of acetone and water under brief anesthesia. Then, the cheek was carefully wiped with ethanol, to eliminate remnants of glue, cream, superficial NPs and capsaicin.

#### 4.7.3. In Vivo Quantification of Capsaicin from PLGA Nanoparticles in Mouse Skin

Skin from mice treated 24 h with NPs was harvested 1 hr after taking off the chamber filled with capsaicin or blank NPs. As a positive control, mice were injected with 30 µg/10 µL of capsaicin in 8% Tween 20 in normal saline (0.9 *w*/*v*) and the cheek skin was harvested 1 hr later. After euthanasia, the treated cheek skin (approximately 1 × 1 cm) was removed and directly frozen in a −80 °C freezer. For analysis, the tissue was cut into small pieces, mixed with 50 µL tissue extraction buffer (20 mM HEPES, pH 7, 420 mM NaCl, 0.2 mM EDTA, 1.5 mM MgCl_2_, 1% NP40, 1 mM PMSF, 25% Glycerol, 0.4% PIC) and homogenized using a Biomasher II^®^ Closed System Disposable Micro Tissue Homogenizer. Samples were centrifuged at 4 °C, 13,000 rpm for 20 min and the supernatant was isolated for further analysis. A Standard Bradford assay was used to measure the total protein concentration of samples. The samples were diluted to an equal total protein concentration of 6 µg/µL for the aforementioned ELISA capsaicin quantification kit in tissue. Three samples per group of mice were analyzed using the ELISA assay.

#### 4.7.4. Analysis of Capsaicin Penetration Depth In Vivo

For the analysis of skin penetration of the NPs, either capsaicin, blank or capsaicin NPs each encapsulated with a dye (DiL) were applied to the cheek of mice for 24 h as described. One hour after cup removal, the cheek was carefully cleaned with 70% ethanol solution. Mice were euthanized under anesthesia and a section of the skin from the cheek was harvested (approximately 1 × 1 cm^2^). The skin was fixated with 4% *w*/*v* PFA/10% formalin for 24 h at −20 °C. Excess PFA was removed by rinsing with PBS and the samples were soaked in 40% *w*/*v* sucrose in PBS for 2 days. For cryoembedding, the tissue was equilibrated at room temperature in OCT-compound for 10 min, transferred and orientated in fresh cooled OCT in a cryomold surrounded by dry ice and isopentane. Samples were stored at −20 °C and cut with a cryostat on the next day into sections of 7 µm thickness. Sample sections were photographed using an EVOS FL Cell Imaging System with a EVOS Light Cube RFP (Thermofisher, Waltham, MA, USA). For statistical comparisons, three images of random sections for six mice per condition were acquired to obtain a mean value. ImageJ software with a color deconvolution plugin [42,43] was used for detecting the percentage of area stained red or red autofluorescence signal compared to the whole tissue sample in the image [44]. Only the epidermis and dermis were included in the analysis and the rest were manually excluded. Colored tissue in each sample was automatically detected using an adaptation to an ImageJ macro, which was designed to count cells in a designated area of the tissue sample [44]. The color deconvoluting macro was used to separate the staining colors in each image. The Focinator tool was used to threshold and binarize the color-separated images, segmenting the stained and unstained regions and differential quantification of intensity for stained areas from each micrograph. The macro automatedly detected regions of interest using a thresholded mask of the image and measuring the different areas across multiple regions of interest per image. Additionally, each image was surveyed for fluorescence in the dermis and epidermis by an operator who was blinded to the treatment conditions.

#### 4.7.5. Testing Mice for Behavioral Responses to Mechanical and Heat Stimulation

Prior to collecting data, each mouse was placed daily for 5 days in a meshed test chamber for 30 min. After 15 min, mechanical and heat stimuli were periodically applied to the cheek as described for testing but with only 5 presentations of each stimulus, always ending with the lowest intensity [45]. The mechanical stimuli consisted of nylon filaments with tip diameter (and delivering different bending forces) of 67 µm (0.23 mN) and 100 µm (2, 10 and 20 mN). A contact thermode with a chip resistor (2 × 3 mm) and a thermocouple were used for electronically servocontrolling temperature at the skin-probe interface, delivering stimulus temperatures of either 38 or 52 °C. 

After the 5 days of habituation to the testing chamber and stimuli, daily testing began 24 h after removal of the Finn chamber. The mechanical stimuli were presented first. Each stimulus force was presented five times and delivered in order of ascending force and again in descending order of applied force. Then five warm, 38 °C stimuli were delivered followed by five presentations of noxious 52 °C stimuli. Lastly, the same heat stimuli were presented again this time with stimuli of 52 °C delivered first. Each mechanical or heat stimulus was applied for a maximal contact time of one second or less if withdrawal occurred and with interstimulus intervals of at least 60 s. 

During testing, mice were video recorded from the side, with a mirror positioned on one side to allow for a two-sided view. After each stimulation, the behavioral responses to each stimulus were documented and later confirmed or corrected in the videorecording. The operator performing the experiments was blinded to the placebo and treatment groups. Each behavioral reaction to a stimulus was assigned according to the following categories; a discomfort score (DS) according to whether “no reaction” (DS = 0), “looking” or turning the head or body towards the stimulating object (DS = 1), “withdrawal’ from the stimulus by turning the head or body away or pulling backward after stimulation (DS = 2), rapid “flinching” (DS = 3), “biting”, consisting of a fast turning of the head toward the object and trying to bite it (DS = 4), “shaking”, a short, quick vibratory movement of the body (DS = 5), “jumping aside” from the stimulus (DS = 6), “jumping in the air” (DS = 7), or audible squeaking (DS = 8). In the case of observing a series of two immediate behaviors, only the behavior with the highest score was used. Each wipe directed towards the stimulated cheek was counted and added to the DS. The mean score from ten presentations of the same stimulus was calculated.

### 4.8. Testing Human Subjects for Responses to Heat, Mechanical Stimuli and to Pruritogens

Twenty-one healthy human subjects (10 females and 11 males, age: 28.9 ± 8.3) were included in this study. Subjects reporting a history of dermatological, neurological, immunological or cardiac disorders were excluded. In addition, subjects were required to refrain from taking antihistamines or analgesics at least 24 h prior to the first experiment and during the whole testing period. All protocols were approved by the Yale University Human Investigative Committee. Before the experiments, participants were trained to use the generalized Labeled Magnitude Scale (gLMS) to rate the perceived intensity of itch and pain-like (nociceptive) sensations of pricking/stinging and burning evoked by a given stimulus [29]. Itch was defined as a sensation that evokes a desire to scratch. Pricking/stinging was defined as a sensation that was sharp and well localized, either intermittent like a needle prick or continuous like an insect sting. Burning was defined as a sensation most often associated with sunburns or thermal burns, but also with skin abrasions, strong cold, or chemical irritants. Participants were instructed that the nociceptive sensations may or may not be painful (“hurt”).

Beginning with the application of a pruritogen and every 30 s thereafter, subjects were instructed to use a computer mouse to make three successive ratings using the gLMS presented on a video-screen using DAPSYS 8 software (http://www.dapsys.net/ accessed on 3 April 2022, Brian Turnquist, Bethel University, St. Paul, MN, USA). The scale consisted of a vertical line with labels, positioned in a quasi-logarithmic manner, of “no sensation”, “barely detectable”, “weak”, “moderate”, “strong”, “very strong”, and “strongest imaginable sensation”. The subject was instructed to judge the maximal magnitude of each of the three qualities of sensation that occurred during the previous interval of 30 s, first “itch”, then “pricking/stinging” and lastly “burning” [46]. Ratings were recorded every 30 s until either there occurred no sensation of any kind for three successive 30 sec periods or after a 20 min period had elapsed. 

The subject’s ratings were saved from the position of the cursor on the scale and converted to a numerical value between 0 and 100. The numbers were not visible to the subject. The positions of the labels were 0 for “no sensation”, 1 for “barely detectable”, 6 for “weak”, 17 for “moderate”, 35 for “strong”, 53 for “very strong”, and 100 for the “strongest imaginable sensation of any kind”. 

Once ratings of a pruritogen were completed, subjects were tested for the presence of any mechanically evoked hypersensitivity (dysesthesia) surrounding the application site. These were defined as (a) alloknesis, itch evoked by stroking the skin with a cotton swab attached to a coping-saw blade that exerted approximately 100 mN of compressional force; or, alternatively, allodynia, if stroking evoked tenderness, (b) hyperalgesia, characterized by a sensation of enhanced pricking pain to a one-second application of a von Frey filament with a tip diameter of 200 μm and exerting a bending force of 80 mN; (c) hyperknesis, defined as enhanced itch to pricking the skin with a von Frey filament having a tip diameter of 50 μm and exerting a bending force of 20 mN. Subjects were instructed to judge only the intensity of the sensation and not the perceived geometric features of the stimulus itself such as how small or “sharp” it was. Additionally, the borders of any skin reactions such as wheal, erythema, or changes in skin appearance such as lighter areas (blanching, white-reaction or vasoconstriction), were marked on the skin following each experiment.

### 4.9. Application of NPs

Following gentle cleaning of the arms with ethanol wipes and taking a picture of the skin, filled Finn chambers with an inner diameter of 12 mm were randomly placed on the volar forearm and held in place with Tegaderm film and additionally covered with white tape. The opposite forearm received the same treatment with Finn chambers filled with blank NPs. The chamber was left in place for four days. Subjects and investigators were blinded to the type of application. The locations of the Finn chambers on the arms were documented and photographed to be able to find the correct areas for further testing. The subjects were asked to refrain from sunbathing, bathing and strenuous physical activity to prevent alterations in the pharmacokinetics of the applied PLGA NPs, and received a cover for showering to ensure the integrity of the Tegaderm. The subjects were provided with four paper copies of the labeled magnitude rating scale to rate the mean and maximal perceived intensity of itch, pricking/stinging, burning, and pain and describe other experienced effects separately for left and right arm. Ratings were performed every hour for the first 8 h. The subjects were asked to continue the ratings in the morning and once in the evening every day for three days. On the day 4, the subjects returned to the laboratory, where the Finn chambers were removed, and the skin was cleaned with alcohol wipes and gently washed with soap and water. 

### 4.10. Ratings of the Perceived Intensity of Pain Evoked by Punctate Mechanical Stimuli of Differing Indentation Force

Von-Frey type filaments having a tip diameter of 200 µm and delivering bending forces of 2, 8, 16, 32, 64, 128, 256 and 512 mN were initially each applied once to the treated area of skin in ascending order of force. The duration of indentation was 1 s and the interstimulus interval 30 s. In response to each stimulus the subject assigned a number of his/her own choosing for a rating in proportion to the perceived intensity of any “pain” sensation (here defined as any painful or pain-like quality such as pricking/stinging) according to the method of magnitude estimation [47]. If there occurred a sensation of touch-pressure with no pain or pain-like quality, the subject assigned the number zero. The stimuli were applied to the treated area of the arm prior to, and again 1, 7, 14 and 21 days after removal of the NPs. The ratings were normalized with respect to each subject’s particular internal modulus as follows [47]. For each subject, the mean of the ratings for every stimulus was obtained and an overall grand mean of these means was calculated across all subjects. By dividing each subject’s mean into the grand mean, a normalizing factor was obtained which, when multiplied by the subject’s mean, equaled the grand mean. This factor, varying for each subject, was multiplied by each of the subject’s ratings to obtain a new set of now normalized data which were then averaged for the subjects for each stimulus applied on each day of testing. 

### 4.11. Pain Threshold for Nociceptive Punctate Mechanical Stimuli of Differing Indentation Force

The “mechanical pain threshold” (MPT) was obtained on each day of testing in response to the same von-Frey filaments with the use of a Quantitative Sensory Testing (QST) protocol [48]. Each filament was applied first in order of ascending force, starting with the lowest value until the sensation of touch-pressure was accompanied by or changed to pain-like pricking or stinging sensation. This force was used as the first suprathreshold value. Then, the whole process was repeated first with the ascending and then descending series of forces until five supra- and five subthreshold values were obtained. The MPT was defined as the geometric mean of these values.

### 4.12. Thresholds for Warmth and Heat-Pain

The warmth detection threshold (WDT) and heat pain threshold (HPT) were determined in response to heat stimuli delivered with a Peltier contact thermode having a 1 cm^2^ contact area and stimulus temperature electronically controlled within to 0.1 °C of the desired value via feedback from a thermocouple located at the skin-thermode interface [36]. The procedure of testing followed the QST protocol and instructions. The thermode was centered on the locus of prior NP application. An increasing ramp of temperature was delivered at 1 °C/s from a base temperature of 32 °C until reaching 50 °C. The thermode was programmed and the subject’s reaction registered with DAPSYS 8 software. Warmth and heat sensations were registered by the subject using a button connected to the computer. Subjects were asked to press the button when a warming sensation was felt (for WDT) and again when the sensation of warmth changed to an additional, pain-like sensation of pricking/stinging or burning (for HPT). The WDT and HPT were calculated as the average threshold temperature of 3 measurements. Next, the subjects were presented with a warm stimulus of 38 °C for 40 s and a following suprathreshold stimulus 50 °C for 6 s. These suprathreshold stimuli were each an incremental heat stimulus with a trapezoidal temperature waveform. After these stimuli, the subjects were asked to rate the perceived magnitude of warmth, burning, pricking/stinging, and pain on the gLMS scale.

### 4.13. Ratings of Perceived Intensity of Itch and Nociceptive Sensations in Response to Pruritogens

**Figure 10:**
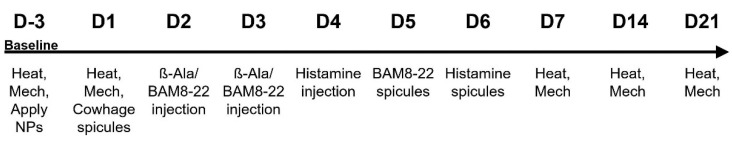
Timeline for human testing. Thresholds for warmth, painful heat and to mechanical stimuli (Mech) were obtained at the test site on each arm prior to application of blank and capsaicin NPs on Day 3 and again after NP removal four days later (D1) and again on D7, 14 and 21. Sensory ratings to cowhage spicules on D1, and to injection of β-alanine (β-Ala), BAM8-22 and histamine as indicated and subsequently to application in heat-inactivated spicules containing BAM8-22 and to histamine.

Following removal of the Finn chambers, MPT, WDT and HPT were determined as on day 3, except that both arms were tested. After a 30 min break, subjects were asked to rate, the itch, pricking/stinging, and burning every 30 s after the application of a pruritogen to each test site. A different pruritogen was applied on each day of testing (Figure 10): day 1, cowhage spicules (trichomes from the plant, *Mucuna pruriens*) [10]; day 2, injection of either 90 mg β-alanine or 1 mg/mL BAM8-22 in 10 µL of normal saline (selected in randomized order and double-blinded); day 3, injection of the other pruritogen (β-alanine or BAM8-22); day 4, injection of 10 µg of histamine in 10 µL of normal saline; day 5, heat-inactivated spicules previously soaked in 3 mg/mL of BAM8-22; day 6, heat-inactivated spicules previously soaked in 10 mg/mL of histamine [10]. After all the sensations evoked by a particular pruritogen disappeared, or 20 min had elapsed, the borders of regions exhibiting any of the dysesthesias to the three mechanical stimuli were mapped on the skin. The mapped arms were photographed along with a scale bar; areas of dysesthesias were analyzed using ImageJ. Ratings of subjects were assessed using DAPSYS 6.

### 4.14. Statistical Analyses

Data evaluation and all analyses were performed by blinded investigators. For each pruritogen applied to the forearm and for each sensory quality (itch, pricking/stinging and burning) a comparison of differences in ratings of a pruritogen over time between blank- and capsaicin-treated areas were analyzed using a within subject repeated measures mixed effect model with Bonferroni post hoc correction.

For each pruritogen and each sensory quality, the means for peak magnitude, AUC and duration were analyzed for differences between the two NP treatments (placebo and capsaicin PLGA NPs) using a Wilcoxon signed-rank test.

Differences between groups in the proportions of subjects reporting a sensation were analyzed with the Fisher’s exact test. Differences between areas of dysesthesias and skin reactions were analyzed with a Wilcoxon matched pairs signed-rank test. Behavioral responses to mechanical and heat stimuli in mice were assessed with a repeated measures two-way ANOVA. For statistical analysis, GraphPad Prism 8 (GraphPad Software, Inc., La Jolla, CA, USA) was applied.

## Data Availability

The datasets used and/or analyzed are available from the corresponding author upon reasonable request.

## Supplementary Materials

The following supporting information can be downloaded at: https://www.mdpi.com/article/10.3390/ijms23095275/s1.
