# Supplementary material for: Topical Capsaicin in Poly(lactic-co-glycolic)acid (PLGA) Nanoparticles Decreases Acute Itch and Heat Pain
Source: Int J Mol Sci. 2022 May 9;23(9):5275. doi: 10.3390/ijms23095275 (PMC9101161; doi:10.3390/ijms23095275)
Supplement: Supplementary file 1 [file ijms-23-05275-s001.zip › ijms-1690556-supplementary.pdf]

International Journal of *Molecular Sciences*

MDPI

Supplementary figures

Topical Capsaicin in Poly(lactic-co-glycolic)acid (PLGA) Nanoparticles Decreases Acute Itch and Heat Pain

Nathalie M. Malewicz [1,2,*], Zahra Rattray [3], Sebastian Oeck [4,5], Sebastian Jung [1,6], Vicente Escamilla-Rivera [1,7], Zeming Chen [8], Xiangjun Tang [8], Jiangbing Zhou [8] and Robert H. LaMotte [1,*]

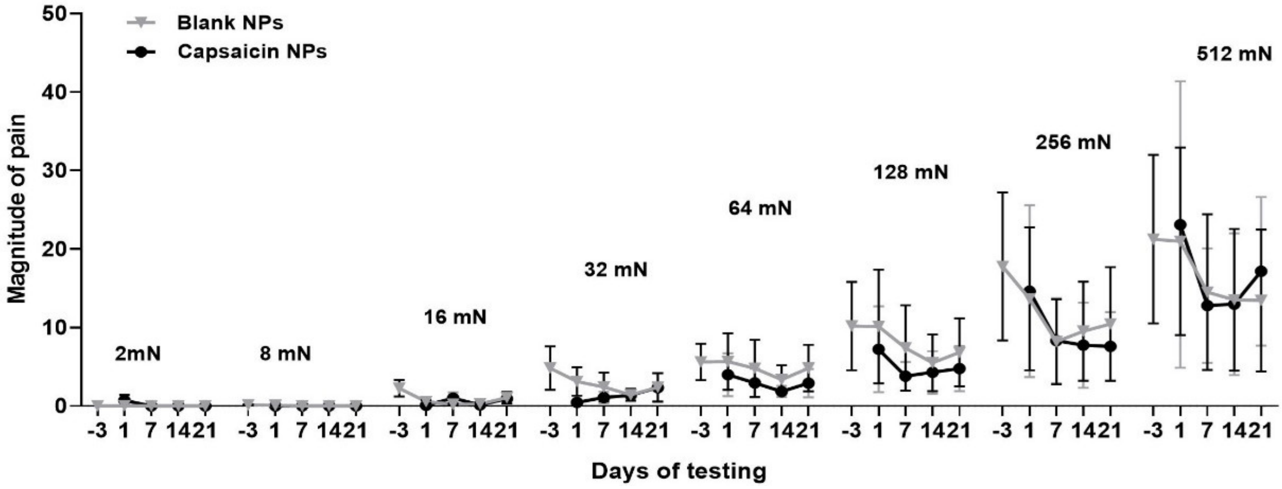

Supplementary Figure S1: Magnitude ratings of pain evoked by mechanical stimuli obtained at sites of blank and capsaicin NPs 4 days before and on each designated day after removal of the NPs. The mechanical stimuli delivered indentation forces of 2 to 512 mN. Mixed effect model 2 within subject treatments (capsaicin vs. placebo treatment) x 4 repeated timepoints (0, 7, 14, 21 days) with Bonferroni correction. Means with SEMs for capsaicin- and blank NPs are black and gray, respectively (n = 21). * $p < 0.05$ and ** $p < 0.01$ for blank vs. NPs comparison.

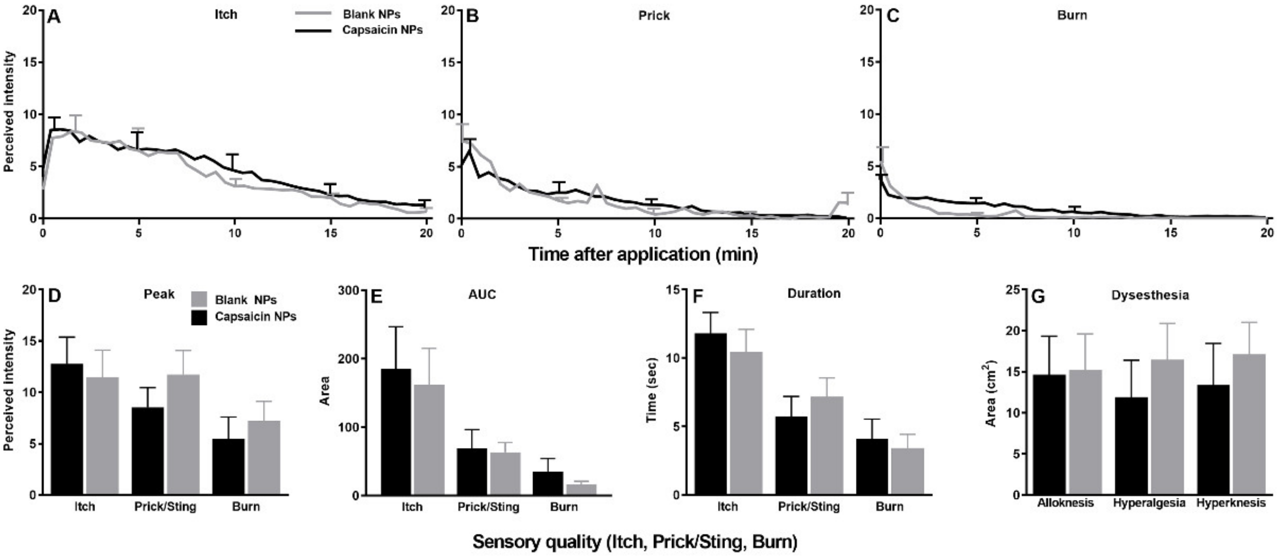

Supplementary Figure S2. Effects of capsaicin NP pretreatment on sensations and dysesthesias evoked by injection of histamine. (**A**-**C**) Time course of perceived intensities of itch, pricking/stinging, and burning. Same format as Figure 7 (**D**) Peak magnitude (Peak) of each sensory quality. (**E**) Area under the rating curve (AUC). (**F**) Duration of response. (**G**) Area of each type of dysesthesia: alloknesis, hyperalgesia and hyperknesis. n = 21, (**A**-**C**) repeated measures two-way ANOVA 2 repeated treatment groups (capsaicin vs. placebo treatment) x 40 repeated timepoints (0 - 20 min) with Bonferroni correction, (**D**-**G**) Wilcoxon test. Data are means with SEMs. * $p < 0.05$, ** $p < 0.01$.

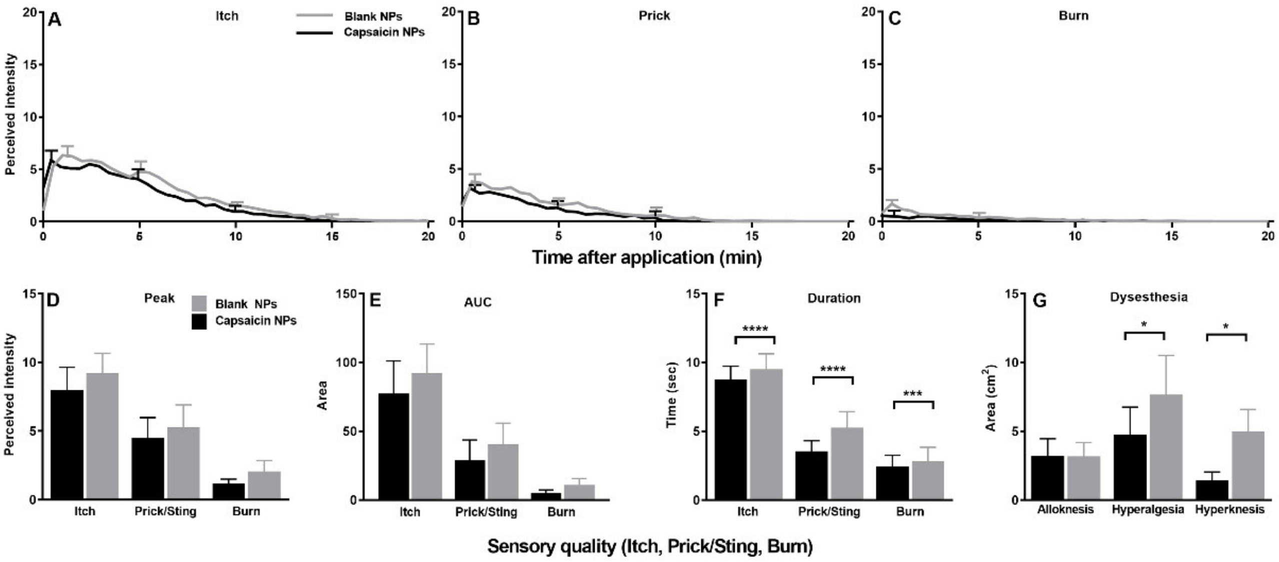

Supplementary Figure S3. Effects of capsaicin NP pretreatment on sensations and dysesthesias evoked by histamine spicules. (**A**-**C**) Time course of perceived intensities of itch, pricking/stinging, and burning. Same format as Figure 7. (**D**) Peak magnitude (Peak) of each sensory quality. (**E**) Area under the rating curve (AUC). (**F**) Duration of response. (**G**) Area of each type of dysesthesia: alloknesis, hyperalgesia and hyperknesis. n = 21, (**A**-**C**) repeated measures two-way ANOVA 2 repeated treatment groups (capsaicin vs. placebo treatment) x 40 repeated timepoints (0 - 20 min) with Bonferroni correction, (**D**-**G**) Wilcoxon test. Data are means with SEMs. * $p < 0.05$, ** $p < 0.01$.
